# Habituation of the Interlimb Reflex (ILR) Over the Biceps Brachii Muscle After Electrical Stimuli Over the Sural Nerve

**DOI:** 10.3389/fnins.2019.01130

**Published:** 2019-10-23

**Authors:** Ssuhir Alaid, Alexander Emmer, Malte Erich Kornhuber

**Affiliations:** Department of Neurology, Martin Luther University of Halle-Wittenberg, Halle (Saale), Germany

**Keywords:** interlimb reflex, electrical train stimulation, facilitation, habituation, upper limb, biceps brachii muscle

## Abstract

Up to now relatively little is known about interlimb reflexes (ILR). Especially it is not well known whether ILR may habituate or not to subsequent stimuli. The main aim of the present investigation was to explore the short term habituation behavior of ILR. The electromyogram was recorded over the tonically active biceps brachii (BB) muscle in 11 healthy subjects contralateral and ipsilateral to supramaximum electrical stimuli (9–12 mA) that were delivered at 1.0 and 0.4 Hz over the left sural nerve. In addition, a selective averaging method was used to investigate the influence of preceding stimuli on the ILR. Thus, 30 blocks of 3 subsequent stimuli were used. All 1st ILR of each block were averaged together. Averages were also obtained for 2nd and 3rd ILR. While ILR amplitudes gained significantly both ipsilateral and contralateral to the stimulus (*p* < 0.05) after train stimuli as compared with single stimuli, ILR amplitudes showed a significant decrease at 1.0 Hz versus 0.4 Hz stimuli. ILR amplitudes decreased significantly after the 2nd and 3rd stimulus relative to the 1st (*p* < 0.05). ILR can be recorded bilaterally remote from the stimulus site. Furthermore, ILR show clear short term habituation behavior.

## Introduction

After electrical limb nerve stimuli, different motor responses can be recorded, such as e.g., H-reflex or the so called long latency reflex (LLR). The H-reflex is a monosynaptic reflex between fast conducting sensory afferent nerve fibers and the alpha motoneurones in a spinal cord segment (Hoffmann, 1910; Táboríková and Sax, 1968). The LLR has been intensively studied in the stimulated limb and seem to be mediated by a transcortical circuit. LLR do not habituate and may even show an incremental behavior after subsequent stimuli (Alaid et al., 2012b). In contrast to LLR, interlimb reflexes (ILR) can be obtained contralateral to the stimulated site and even in the legs when the stimulus was placed over an arm nerve and vice versa (Zehr et al., 2001; Butler et al., 2016). An important feature to discriminate reflex responses is whether they show short term habituation or not. The motor response obtained over the biceps brachii (BB) muscle contralateral to an arm nerve stimulus has been shown to habituate (Alaid et al., 2012a). Neuronal short-term habituation (STH) is regarded as the decrease in transsynaptic responses observed during repeated stimulation at regular time intervals (for review see Fischer et al., 2014). Habituation behavior has also been found to be an important feature of startle reflexes (Davis, 1970). Therefore, our previous results of arm muscle response habituation to contralateral stimuli have been interpreted to be a startle response. Alternatively, it could be that this reflex corresponds to an ILR. It is, however, not clear whether ILR habituate after repetitive stimuli. The main aim of the present study was to clarify whether ILR in general would habituate and could eventually be correlates of startle responses.

## Subjects and Methods

Eleven healthy subjects [hospital staff, 18–45 years (28.1 ± 6.3), 6 females] volunteered in the following experiments, which started at 10 a.m. and lasted about 1.5 h. The subjects gave their informed consent in accordance with the Declaration of Helsinki to participate in the investigation. Ethical approval for the study was received from the Martin Luther University of Halle-Wittenberg. Subjects were laid comfortably on their back. The subject’s right and left forearm were positioned actively bent at an angle of 90° to the upper arm. Surface electromyogram was obtained over the middle of the belly of the short head of the voluntarily contracted BB muscle of right and left arm (contralateral and ipsilateral to the stimulus site) versus the olecranon as reference (Multiliner Economy, Viasys, Höchberg, Germany). Supramaximum rectangular current pulses (9–12 mA) of 0.2 ms duration were delivered over the left sural nerve lateral to the Achilles tendon above the lateral malleolus through two surface electrodes at a fixed distance of 2.5 cm, the cathode set proximal to the anode. “Supramaximum” means that the stimulus strength is that high that all axons in the stimulated nerve are recruited to build up action potentials. Stimulus strength may not be further increased by simply increasing the stimulus current. This would not result in recruiting more nerve fibers to fire. Therefore, train electrical stimuli were used. These consist of single electrical stimuli that are repeated with minute time intervals of e.g., few milliseconds. Beside single stimuli, trains of 2, 3, 4, and 5 stimuli were used with a within-train inter-stimulus interval (ISI) of 3 ms. The flexible wet ground electrode was tied 10 cm above the stimulus site. The time window was 500 ms, 50 ms before stimulus onset and 450 ms thereafter. The recorded signal was visualized at 100 μV/division. Filters were set at 2 Hz, 1 kHz with a sample rate of 5 kHz. No rectification was done since it has previously been shown to be potentially deleterious in LLR recordings over arm muscles due to an excess in phase cancellation (Alaid and Kornhuber, 2013).

**FIGURE 1:**
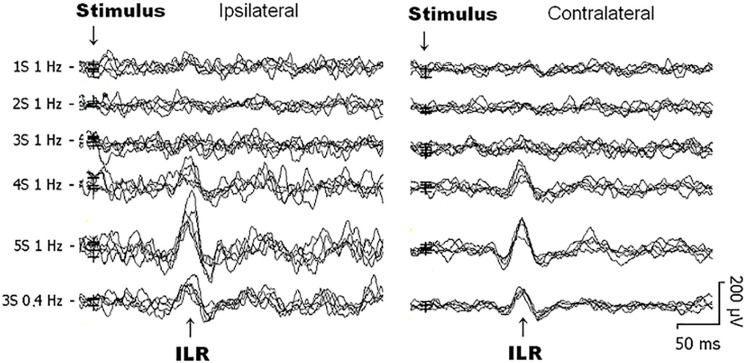
Original interlimb reflex (ILR) recordings obtained over the biceps brachii (BB) muscle ipsilateral **(left)** and contralateral **(right)** to sural nerve stimuli from 1 subject within the same recording session. Note that the ILR is obtained on either side with similar latency times. ILR responses gain in amplitude when the number of single stimulus components within trains is increased. When trains-of-3 are repeated at 1 Hz, ILR amplitudes are considerably smaller as to 0.4 Hz stimuli, presumably due to habituative influences (see Figure 2E).

Two different experimental protocols were run during the same session with the same eleven subjects. The 1st experiment was devoted to the influence on ILR by (i) single and train stimuli, and by (ii) the stimulus repetition rate, i.e., 1.0, and 0.4 Hz (see Figure 1). At least three average curves, each consisting of 30 traces have been obtained in order to assure reproducibility. All average curves of the same condition were taken to form a “secondary” average curve. Evaluation was done using such secondary average curves of 1 Hz single stimuli, double stimuli and trains of 3, 4, and 5 stimuli. For trains of 3 stimuli beside 1 Hz also 0.4 Hz stimuli were employed.

The purpose of the 2nd experiment was to study the influence of a preceding stimulus on the ILR. Blocks of 3 subsequent trails followed by a recovery-break of 10 s were repeated 30 times. Each trail contained a train of 3 stimuli with an interstimulus interval of 3 ms. Train stimuli were delivered at a rate of 1 Hz.

Thirty individual traces obtained after each first of 3 consecutive stimuli were grouped manually on screen and added by mouse click to the according average curve. Similarly, selectively averaged curves were obtained for the responses after the 2nd and 3rd stimulus, respectively.

**FIGURE 2:**
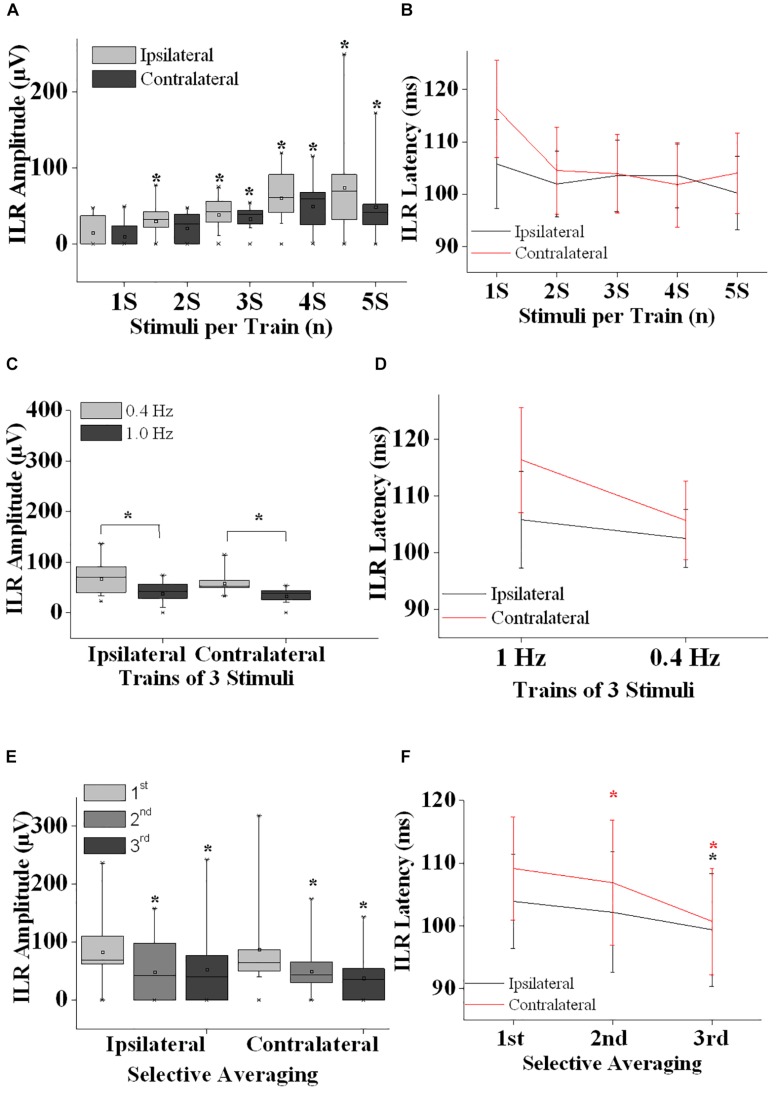
Interlimb reflex results are summarized. **(A)** ILR amplitudes ipsilateral (gray boxes) and contralateral (black boxes) after sural nerve stimuli are shown after single stimuli (1S), double stimuli (2S) and trains of 3–5 stimuli (3S, 4S, and 5S). Note that ILR gain in amplitudes significantly when more than a single stimulus is presented. ILR amplitudes tended to be larger ipsilateral as compared to contralateral to the stimulus site. **(B)** ILR peak latency values are given for the stimulus conditions as shown in panel **(A)**. ILR peak latency values tended to be longer contralateral to the stimulus site **(D,F)**. **(C)** ILR amplitude values ipsilateral and contralateral to trains-of-3 over the sural nerve given at 1.0 and 0.4 Hz. Note that ILR amplitudes were significantly lower both ipsilateral and contralateral after 1.0 Hz stimuli as compared to 0.4 Hz stimuli. This difference in ILR amplitudes is in line with habituative influences **(E)**. **(D)** The ILR peak latency values measured for the stimulus conditions given in panel **(C)**. Note that peak latency values tended to be slightly longer contralateral as compared to ipsilateral to the stimulus site. **(E)** ILR amplitude values are given ipsilateral and contralateral to trains-of-3 delivered over the left sural nerve. ILR responses were averaged selectively after the 1st, 2nd, and 3rd of 3 stimuli that were repeated at a rate of 1 Hz followed by a recovery time interval of 10 s. Note that ILR amplitudes displayed a significant decline in amplitude after the 2nd and 3rd stimulus relative to that of the 1st stimulus, both ipsilateral and contralateral to the stimulus site. This amplitude decline is interpreted to result from habituative influences. **(F)** ILR peak latency values corresponding to the stimulus conditions shown in panel **(E)**. Note that ILR peak latency values decreased after the 2nd and 3rd stimulus relative to the ILR obtained after the 1st stimulus. This difference in peak latency values reached statistical significance ipsilateral and contralateral to the stimulus site for the ILR after the 3rd stimulus while after the 2nd stimulus the decline was significant only contralateral. Note that ILR peak latency values tended to be longer contralateral as compared to ipsilateral to the stimulus site. ^∗^, *p* at least <0.05.

Peak latencies were obtained on screen. Furthermore, peak-to-peak amplitudes were measured. In all experiments, the mean value and the standard deviation (SD) were calculated. With a low number of 11 subjects, normal distribution of the measured values cannot be assumed. Therefore, non-parametric statistical analyses were used instead. As different experimental conditions were investigated at identical recording sites in the same subject, analysis of variance for related sample statistics (Friedman test) were employed to test for statistical significance (*p* < 0.05) with Wilcoxon’s matched pairs test *post hoc* (*p* < 0.05). The following tests were performed: In the 1st experiment all experimental conditions (illustrated in Figure 2, i.e., from ipsilateral and contralateral recordings sites) have been introduced in the Friedman test. Thereafter, data from the ipsilateral site and the contralateral site were investigated by Friedman test separately. Afterward, results obtained after single stimuli were compared with those after train stimuli for ipsilateral data and for contralateral data (Figures 2A,B). For trains-of-3, 1 and 0.4 Hz results were compared after the 1st, 2nd, and 3rd stimulus (Wilcoxon test) ipsilateral and contralateral to the stimulus separately (Figures 2C,D). In the second experiment Friedman test was used to test for significance of all data (ipsilateral and contralateral). Then Friedman test was used to test ipsilateral data and contralateral data separately. Afterward, data after a 2nd and 3rd stimulus were compared with those after a 1st stimulus (Figures 2E,F). Statistica for Windows (StatSoft, version 4.5) was used for statistical analyses.

## Results

### First Experiment

Generally, ILR were of low amplitude (Figures 1, 2A,C) and results were well reproducible for a given experimental condition intraindividually (Figure 1). After sural nerve stimuli, ILR were seen over the BB on either side in all 11 subjects when trains of 3 stimuli were delivered at a rate of 0.4 Hz (Figure 1, 6th trace from top, Figure 2C). When trains of 3 stimuli were given at a rate of 1 Hz, ILR amplitudes were significantly lower as compared to the 0.4 Hz condition (*p* < 0.005 both ipsilateral and contralateral to the stimulus site; Figure 1, traces 3 and 6 from top; Figure 2C). After trains of 3 stimuli at 1 Hz, two subjects did not display a response, one ipsilateral and one contralateral to the stimulus site. Since ILR were present in the majority of the subjects even after 1 Hz stimulus repetition, the influence of stimulus strength was investigated with the more rapid stimulus repetition rate of 1 Hz. After trains of 5 stimuli, 10 subjects showed unequivocal ILR, while after single stimuli, four subjects showed ILR ipsilateral to the stimulus site and three contralateral. The statistical analyses revealed a significant influence of stimulus strength on ILR amplitude values (*p* < 0.00002 ipsilateral to the stimulus, *p* < 0.00002 contralateral; Friedman test). There was roughly a linear and in most cases significant increase in ILR amplitudes from single stimuli up to trains of 5 stimuli, ipsilateral as well as contralateral to the stimulus site (Figure 2A) (*p* < 0.05; Wilcoxon test; as compared with single stimuli). No clear difference in ILR amplitudes was noted, however, when trains of 5 stimuli were compared with trains of 4 stimuli (Figure 2A). ILR amplitudes ipsilateral to the stimulus site were significantly larger as compared to the contralateral side, namely when the values obtained after all 5 stimulus conditions were treated together (*p* < 0.001).

Interlimb reflexes peak latency values were usually distributed between 95 and 120 ms (Figures 2B,D). Friedman test revealed significant alterations when the values from all conditions (ipsilateral and contralateral) were included (*p* < 0.0001). When the ILR peak latency values from all stimulus conditions were taken together, values contralateral to the stimulus site were slightly longer (105.4 ± 8.2 ms) than ipsilateral (102.4 ± 7.1 ms; *p* < 0.005, Wilcoxon test). ILR peak latency values did not show a clear change with the number of stimuli per train (Figure 2B). Presumably by chance, the difference between ILR peak latency values ipsilateral to the stimulus site obtained after trains-of-5 as compared with trains-of-3 reached statistical significance (*p* < 0.05; Wilcoxon-test). No statistically significant changes were found when ILR latency values obtained at 1.0 Hz were compared with those obtained at 0.4 Hz (Figure 2D).

### Second Experiment

On either side, ILR displayed a steady and significant decline from one stimulus to the next when amplitudes after the 1st of 3 subsequent stimuli were compared with those after the 2nd and 3rd stimulus (*p* < 0.02 ipsilateral to the stimulus site and *p* < 0.005 contralateral to the stimulus site, Friedman test; Figure 2E). ILR amplitudes after the 2nd stimulus were significantly lower compared with those after the 1st stimulus, *p* < 0.02 ipsilateral and *p* < 0.01 contralateral to the stimulus site (Wilcoxon test). The respective values for the amplitudes after the 3rd as compared with the 1st stimulus were *p* < 0.005 ipsilateral and *p* < 0.01 contralateral.

The according ILR peak latency values obtained after subsequent stimuli also showed significant changes, namely *p* < 0.02 ipsilateral to the stimulus site and contralateral *p* < 0.005 (Friedman test; Figure 2F). When ILR latency values after the 2nd stimulus were compared with those after the 1st stimulus, there was no significant difference ipsilateral to the stimulus site (Wilcoxon test), while contralateral, the difference was significant (*p* < 0.05). ILR peak latency values after the 3rd were significantly decreased relative to the 1st stimulus on either side (*p* < 0.05).

## Discussion

Following sural nerve stimuli, motor responses have been recorded over the BB muscle on either side in all subjects. Peak latency values of these motor responses were quite similar to what has been reported for ILR in healthy persons or in control subjects previously, namely in the range of 100 ms (Zehr et al., 2001; Butler et al., 2016). This similarity in the latency values makes it likely that the observed motor responses in our healthy subjects indeed reflect ILR. While in our experiments, ILR latency values varied considerably between different subjects (Figures 2B,D), this variation was rather small intraindividually, when all different experimental conditions from either side were compared (2.6 ± 1.2 ms, mean ± SD of the differences between maximum and minimum individual peak latency values ipsilateral and contralateral to the stimulus site). Thus, the interindividual variation of ILR latency values in the range of 88–124 ms ipsilateral and contralateral to the stimulus site may reflect interindividual differences in distances between stimulus and recording sites. Also variations in nerve fiber conduction between subjects may contribute to the results. The conduction delay of on average 3 ms from one side of the spinal cord to the other presumably takes place on a segmental spinal cord level. The reason for this delay is unclear at present. The delay could either result from an interneuron or merely from a longer pathway when poorly myelinated fibers are taken into consideration. The contribution of weakly myelinated nerve fibers has already been discussed in the context of ILR before (e.g.,Butler et al., 2016).

Interlimb reflexes peak latency values were shorter with the faster stimulus repetition rate of 1 Hz as compared to the slower stimulus repetition rate of 0.4 Hz (Figure 2D, not significant). This difference could not be accounted for by the influence of preceding stimuli. Thus, in the 2nd experiment, a significant shortening of ILR peak latency values was seen on either side after the 3rd as compared with after the 1st stimulus (Figure 2F). Reason, meaning and relevance of these opposite changes of ILR peak latency values remain unclear.

Reflex responses in the nervous system segregate in those without short term habituative influences such as the H-reflex or the LLR (e.g., Alaid et al., 2012b) and those with habituative influences such as startle reflexes or as the medium latency reflex (Alaid et al., 2014). In the present study, the ILR over the BB muscles after sural nerve stimuli showed a significant decremental response on both sides from one stimulus to the next (2nd experiment, Figure 2E). When conventional averaging was used, the habituative influence is clearly visible as a significantly lower ILR amplitude after 1 Hz stimulus repetition (trains-of-3) as compared with 0.4 Hz stimulus repetition (trains-of-3), (1st experiment, Figure 2C). These results indicate that habituative influences mediate the amplitude decline. Thus, the ILR seems to be subject to inhibitory interneurons that lead to hyperpolarization of ILR target neurons depending on the stimulus repetition rate.

Almost simultaneous ILR-appearance all over the body does not well fit when a function in coordinate limb movements e.g., during walking or running (Zehr et al., 2001) or as a priming for stumbling corrections or for falling preparations is taken into consideration (Zehr and Duysens, 2004). The ubiquitous presence and its habituation behavior set the ILR in the vicinity of the group of the startle reflexes. Previously it was shown that motor responses over cranial and cervical muscles could merely be eliminated with a weaker prepulse to a subsequent stronger stimulus e.g., over the median nerve. At the same time, the responses over wrist flexor muscle of the stimulated limb persisted (Álvarez-Blanco et al., 2009). This persistence does not well fit to a brainstem mediated startle reflex and has been discussed in the context of a possible withdrawal reflex (Álvarez-Blanco et al., 2009). As far as we can see, withdrawal reflexes are most prominent in the stimulated limb (Andersen, 2007). In our investigation we got bilateral responses over muscles remote from the stimulus site (BB muscles after unilateral sural nerve stimuli). These remote responses are presumably not withdrawal reflexes. Taken together, the motor responses in our study seem to be ILR, and their habituation behavior is similar as in medium latency reflexes (Alaid et al., 2014) or as in startle reflexes. Yet, presumably the ILR in our study are not generated from brainstem sites as similar responses have been recorded in subjects with high cervical spinal cord transaction (Calancie, 1991; Zehr and Duysens, 2004).

Interlimb reflexes could be easily evoked in healthy subjects over actively contracted muscles (Zehr et al., 2001), while they could be less well evoked over relaxed muscles (Butler et al., 2016). Similar influences of volition on the magnitude of reflex responses have previously been studied in different reflex responses from the central nervous system (Alaid et al., 2012b, 2014).

Train stimuli have been employed to elicit ILR (Zehr et al., 2001; Butler et al., 2016). In the present investigation, ILR gained significantly in amplitudes depending on the number of stimuli per train (Figure 2A). Actually, the gain in amplitude on average reached a maximum when the trains were composed of 4 stimuli while there was no further increase in ILR amplitudes when trains-of-5 were used (Figure 2A). The influence of the number of stimuli per train on ILR size is presumably due to temporal summation of the resulting excitatory postsynaptic potentials at the involved target neurons. Similar effects have been observed when other reflex responses such as e.g., LLR have been in the scope (Conrad and Aschoff, 1977; Alaid et al., 2012b).

## Data Availability Statement

All datasets generated for this study are included in the manuscript and/or the supplementary files.

## Ethics Statement

Ethical approval for the study was received from the Martin Luther University of Halle-Wittenberg.

## Author Contributions

SA and AE conceived of the presented idea. SA developed the theory and performed the computations. AE and MK verified the analytical methods. MK encouraged SA to investigate the Interlimb reflex and supervised the findings of this work. All authors discussed the results and contributed to the final manuscript. SA wrote the manuscript with the support from AE and MK.

## Conflict of Interest

The authors declare that the research was conducted in the absence of any commercial or financial relationships that could be construed as a potential conflict of interest.
